# Resveratrol as a potential protective compound against skeletal muscle insulin resistance

**DOI:** 10.1016/j.heliyon.2023.e21305

**Published:** 2023-10-30

**Authors:** Arash Bahramzadeh, Kosar Bolandnazar, Reza Meshkani

**Affiliations:** a Department of Clinical Biochemistry, Faculty of Medicine, Tehran University of Medical Sciences, Tehran, Iran; b Department of Biological Sciences and Technology, Islamic Azad University of Mashhad, Mashhad, Iran

**Keywords:** Resveratrol, Skeletal muscle, Inflammation, Oxidative stress, AMPK, SIRT1, Insulin resistance, Lipid accumulation

## Abstract

The increasing prevalence of type 2 diabetes has become a major global problem. Insulin resistance has a central role in pathophysiology of type 2 diabetes. Skeletal muscle is responsible for the disposal of most of the glucose under conditions of insulin stimulation, and insulin resistance in skeletal muscle causes dysregulation of glucose homeostasis in the whole body. Despite the current pharmaceutical and non-pharmacological treatment strategies to combat diabetes, there is still a need for new therapeutic agents due to the limitations of the therapeutic agents. Meanwhile, plant polyphenols have attracted the attention of researchers for their use in the treatment of diabetes and have gained popularity. Resveratrol, a stilbenoid polyphenol, exists in various plant sources, and a growing body of evidence suggests its beneficial properties, including antidiabetic activities. The present review aimed to provide a summary of the role of resveratrol in insulin resistance in skeletal muscle and its related mechanisms. To achieve the objectives, by searching the PubMed, Scopus and Web of Science databases, we have summarized the results of all cell culture, animal, and human studies that have investigated the effects of resveratrol in different models on insulin resistance in skeletal muscle.

## Graphical abstract

**Figure:**
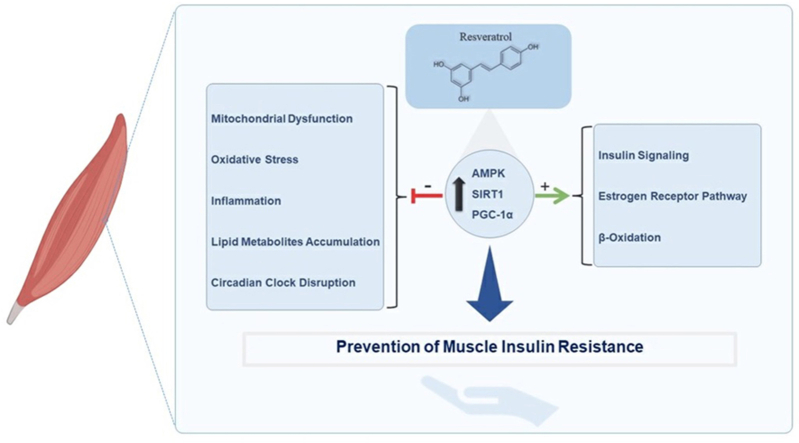

## Abbreviations

ACC2: Acetyl-CoA carboxylase 2
AICAR: 5-aminoimidazole-4-carboxamide 1-β-d-ribofuranoside
AMPK: AMP-activated protein kinase
ANT2: Adeninenucleotide translocase 2
ATM: Ataxia-telangiectasia mutated
BMAL1: Brain and muscle Arnt-like protein-1
CaMKK: Calcium/Calmodulin-dependent Protein Kinase Kinase
CAV-3: Caveolin 3
CLOCK: Circadian locomotor output cycles kaput
cPKC: Conventional protein kinase C
CRY: Cryptochrome, CUG: Catch-up growth
DAGs: Diacylglycerols
DPP-4: Dipeptidyl Peptidase IV
EGCG: Epigallocatechin Gallate
ER: Estrogen receptor
ER stress: Endoplasmic Reticulum stress
ETC: Electron transport chain
FFA: Free fatty acid
GDM: Gestational diabetes mellitus
GIV/Girdin: Gα-interacting,Vesicle-associated Protein/Girdin
GK: Goto-Kakizaki
GLUT4: Glucose transporter type 4, GSK-3: Glycogen synthase kinase 3
GSV: Glucose transporter storage vesicle
H3K9me3: Methylation of Lysine 9 in Histone H3
HDAC3: Histone deacetylase 3
HFD: High Fat Diet
HMB: β-Hydroxy-β-methylbutyrate
IKKβ: Inhibitor of nuclear factor kappa-B kinase subunit β
IL: Interleukin
IMCL: Intramyocellular lipid
IMF: Intermyofibrillar
iNOS: Inducible nitric oxide synthase
INSR: Insulin Receptor
IRE1α: Inositol-requiring enzyme 1α
IRS-1: Insulin receptor substrate 1
IUGR: Intrauterine Growth Restriction
IκBα: Nuclear factor of kappa light polypeptide gene enhancer in B-cells inhibitor
α, JNK: c-Jun N-terminal Kinase
LCFA-CoA: Long-chain fatty acyl-CoA
LKB1: Liver Kinase B1
LPS: Lipopolysaccharide
MAPK: Mitogen-activated protein kinase
MCP-1: Monocyte Chemoattractant Protein-1
MDA: Malondialdehyde
MEF2A/D: Myocyte-specific enhancer factor 2 A and D
mTOR: Mammalian target of rapamycin
NAD: Nicotinamide adenine dinucleotide
NAFLD: Nonalcoholic fatty liver disease
NF-κB: Nuclear factor kappa-light-chain-enhancer of activated B cells
NO: Nitric oxide
nPKC: Novel Protein kinase C
NR1D: Nuclear receptor subfamily 1 group D
PCOS: Polycystic ovary syndrome
PDK1: Phosphoinositide-dependent kinase 1
PER: Period
PERK: Protein kinase RNA-like endoplasmic reticulum kinase
PGC-1α: Peroxisome proliferator-activated receptor γ coactivator 1-α
PI3K: Phosphoinositide 3-kinase
PKC: Protein kinase C
PP2A: Protein Phosphatase 2A
PPAR: Peroxisome Proliferator-Activated Receptor
PTP1B: Protein Tyrosine Phosphatase 1B
RBP4: Retinol binding protein 4
ROR: Retinoid-related Orphan Receptor
ROS: Reactive oxygen species
SFA: Saturated fatty acid
SGLT2: Sodium-Glucose Transport Protein 2
SIRT1: Sirtuin 1
Slc2a4: Solute carrier family-2-member-4
SNAP: Synaptosome associated protein
SNARE: Soluble N-ethylmaleimide-sensitive factor attachment protein receptor
SS: Subsarcolemmal
STZ: Streptozotocin
TBC1D4: TBC1 Domain Family Member 4, or Akt substrate of 160 kDa (AS160)
TCA: Tricarboxylic acid
TLRs: Toll-like receptors
TNF-α: Tumor necrosis factor α
TRB3: Tribbles pseudokinase-3
TUG: Tether containing a UBX domain for GLUT4
TZDs: Thiazolidinediones
UPR: Unfolded protein response
VAMP2: Vesicle associated membrane protein 2
ZDF: Zucker diabetic fatty

## 1 Introduction

One of humanity's critical and worrying challenges in the 21st century is the growing prevalence of diabetes. Based on 2021 International Diabetes Federation estimates, it is predicted that by 2045, 783 million people will be diagnosed with diabetes, representing a 46 % growth rate [1]. The pathogenesis of type 2 diabetes includes the combination of insulin resistance and β cell dysfunction [2]. Insulin resistance is associated with a variety of clinical conditions, such as prediabetes, non-alcoholic fatty liver disease (NAFLD) [3], lipodystrophy [4], and poly ovary syndrome (PCOS) [5,6]. Although the causes of type 2 diabetes have not been precisely identified, obesity is considered the most important risk, along with other factors such as age, ethnicity, and family history. Insulin resistance in skeletal muscle plays a crucial role in the pathogenesis type 2 diabetes. Skeletal muscle, as the largest organ in the body (constitutes about 40 % of body mass), is responsible for approximately 80 % of postprandial glucose uptake from the bloodstream during insulin stimulation under normal conditions. By stimulating insulin, the skeletal muscle uptakes, storages, and utilizes glucose and, along with the liver and adipose tissue, moves the body towards maintaining and regulating the blood glucose level. Any skeletal muscle dysfunction affects glucose homeostasis in the whole body and leads to impaired glucose tolerance and type 2 diabetes [[7], [8], [9]].

Pharmacological and non-pharmacological treatment strategies have the potential to improve insulin sensitivity and increase the chances of survival in patients with diabetes. Over the last few decades, a great deal of attention has been focused on using natural polyphenols as modern therapies to treat insulin resistance and diabetes [10]. Polyphenols are a major group of bioactive compounds with a common phenolic structure composed of hydroxyl groups on the aromatic ring. They are naturally present in various plants as secondary metabolites and play an important role in adapting plants to their environment. In recent years, extensive research has been conducted on polyphenols derived from vegetables, fruits, grains, dry legumes, cocoa, and plant beverages, including coffee, tea, and wine, indicating their beneficial role in health and preventing or improving different types of diseases [11].

Resveratrol (3, 5, 4′ trihydroxystilbene; RSV), is a natural diphenolic compound that is abundant in grape skins and seeds, as well as in plant foods and beverages such as peanuts, berries family and red wine [12,13]. One of the limitations of using resveratrol as a commercially available supplement is its low bioavailability and fast metabolism rate. After consumption of resveratrol, about 70 % of this compound is absorbed in the digestive system primarily by passive diffusion. However, it is rapidly metabolized in the liver and intestine through three different pathways in the form of glucuronide, sulfate, or hydrogenation of aliphatic double bonds. The bioavailability and pharmacokinetics of resveratrol depends on the dosage, dietary matrix co-administration, particle size, gut microbiota, and circadian changes [11,14]. Extensive studies have shown the pleiotropic therapeutic effects of resveratrol, such as anti-obesity [15], anti-cancer [[16], [17], [18]], cardiovascular protective [[19], [20], [21]], anti-neurological [[22], [23], [24]], anti-diabetic [25,26], anti-oxidant [27,28], anti-inflammatory [29,30], anti-platelet aggregation [31], and anti-atherogenic [32] properties in vitro and in vivo studies [33].

Studies on the effects of resveratrol on the improvement of hyperglycemia and insulin resistance have been of great interest to researchers, but whether resveratrol is effective in improving insulin resistance in skeletal muscle or not has been debated. Therefore, the purpose of the current review article is to review the effects of resveratrol on insulin resistance in skeletal muscle tissue and the possible mechanisms by which resveratrol may exert its effects. For this purpose, we searched PubMed, Scopus and Web of Science databases for reports of studies on resveratrol, skeletal muscle, and type 2 diabetes/insulin resistance with different combinations. This paper first gives a brief overview of the mechanisms involved in the development of insulin resistance in skeletal muscle. Cell and animal model studies will be examined in the second and third sections. The final section summarizes the principal findings of clinical trial studies.

## 2 Molecular mechanisms underlying insulin resistance in skeletal muscle

The molecular mechanisms causing insulin resistance in skeletal muscle have been previously reviewed in detail [2,[34], [35], [36], [37], [38], [39], [40], [41]]. Here, we summarize this issue for better understanding of the readers.

### 2.1 1: Insulin signaling impairment

GLUT4, a key regulatory protein for glucose transport, is essential to the glucose uptake of skeletal muscle cells [42]. Insulin exerts its biological effects by binding to its tyrosine kinase insulin receptor (INSR) on the cell and activating two signaling pathways, namely phosphoinositide 3-kinases (PI3K)/Akt, which plays a role in regulating cell metabolism, and RAS- mitogen-activated protein kinase (MAPK), which plays a role in cell growth and proliferation [36]. Tyrosine phosphorylation of the insulin receptor substrates (IRS)-1 and 2 by INSR activates serine/threonine kinase Akt via PI3K activation. Through the phosphorylation of several substrates in its downstream pathway, Akt increases the expression and translocation of intracellular vesicles containing GLUT4 to the myocyte membrane and on the other hand, through the inactivation of glycogen synthase kinase 3 (GSK-3), it stimulates glycogen synthesis in the myocytes [43]. In insulin resistance state, the downregulation of the PI3K/Akt pathway and the upregulation of RAS-MAPK can be observed [44]. The insulin signaling defect results in a reduction in glucose transport. GLUT4 levels have been shown to be significantly reduced in skeletal muscle of patients with type 2 diabetes as well as in subjects with insulin resistance [45].

### 2.2 Mechanisms linking lipid accumulation and insulin resistance

Intramyocellular lipid (IMCL) accumulation is inversely related to insulin sensitivity in skeletal muscle because it can disrupt the insulin signaling pathway [46]. High circulating free fatty acid (FFA) levels during intravenous lipid infusion, obesity, insulin resistance, and type 2 diabetes lead to FFA oversupply to muscle and IMCL accumulation [47]. IMCL refers to any form of lipid stored within myocyte lipid droplets, mainly triglycerides and sometimes lipid metabolites such as diacylglycerols (DAGs), ceramides, and long-chain fatty acids-CoA (LCFAs-CoA). The evidence shows that the intracellular accumulation of triglycerides is not the main factor contributing to insulin resistance in skeletal muscle, but it appears that the accumulation of lipid metabolites such as DAGs, ceramides, and LCFAs-CoA have important role in disruption of the insulin signaling pathway [48,49]. DAGs can activate protein kinase C θ (PKCθ), a novel isoenzyme of PKC (nPKC), in skeletal muscle. PKCθ, by serine phosphorylation of IRS-1, PDK1, and guanine exchange factor GIV/Girdin and inhibiting them, reduces PI3K/Akt signaling [49]. Ceramides reduce the PI3K/Akt pathway by activating protein phosphatase 2A (PP2A) and disrupting Akt signaling through PKCζ activation [50]. Also, PKCs can trigger inflammatory signals by activating the inhibitor of nuclear factor kappa B kinase subunit beta (IKKB) and c-Jun NH2-terminal kinase (JNK), as will be reviewed below.

### 2.3 The role of inflammation in skeletal muscle insulin resistance

Low-grade chronic inflammation caused by obesity is related to the occurrence of diabetes and is regarded as one of the major causes of insulin resistance in skeletal muscle. As a result of obesity, circulating pro-inflammatory cytokines originating from adipose tissue and infiltrated macrophages, are increased [51,52]. These cytokines such as tumor necrosis factor α (TNF-α), interleukin β (IL-1β), and IL-6, can have endocrine effects on skeletal muscles [53]. Moreover, like adipose tissue, skeletal muscle can secrete factors called myokines and can also be a site of infiltration of immune cells, in particular, macrophages with M1 pro-inflammatory phenotype. M1 macrophages residing in skeletal muscle produce inflammatory cytokines, which with autocrine and paracrine effects, can further induce inflammatory responses in skeletal muscle [53]. In addition, increasing the supply of FFAs to skeletal muscle following obesity through increasing the ectopic accumulation of IMCL metabolites in myocytes and also by the activation of toll like receptors (TLRs) can promote inflammatory pathways in skeletal muscle [54]. Finally, the inflammatory responses created in skeletal muscle following the activation of TLRs, cytokine receptors, and the accumulation of lipid metabolites is associated with the activation of NF-kB and JNK pathways, which consequently can interfere with the insulin signaling via serine/threonine phosphorylation of IRS-1 and INSR [55,56].

### 2.4 Role of mitochondrial dysfunction in skeletal muscle insulin resistance

Mitochondrial dysfunction plays an important role in the pathogenesis of insulin resistance in skeletal muscle [57]. Mitochondrial dysfunction is determined by reducing the activity of enzymes involved in mitochondrial activity (such as FFA oxidation, tricarboxylic acid cycle cycle, and respiratory chain components), altering morphology, decreasing the number of mitochondria (the balance between biogenesis/degradation or fusion/fission) and consequently, decrease in the oxidative capacity and intrinsic function of mitochondria [58]. The decrease in mitochondrial function can be caused by hereditary or acquired factors such as lifestyle, a high-fat diet (HFD), and aging. The decrease in mitochondrial function or number leads to a reduction in the rate of β-oxidation of FFAs, and this dysregulation of β-oxidation of FFAs can increase the accumulation of IMCL and lipid metabolites and thereby disrupt the insulin signaling, as explained above [59]. In addition, reactive oxygen species (ROS) produced as a result of altered mitochondrial respiratory function due to increased supply over demand of energy for oxidation (e.g., under high-fat diet conditions), also contribute to insulin resistance through different mechanisms [60].

### 2.5 The impact of oxidative stress on muscle insulin resistance

Oxidative stress derived from high concentrations of ROS, such as superoxide anion radical (O2•-), hydroxyl radical (•OH), and hydrogen peroxide (H_2_O_2_), is another mechanism involved in the pathogenesis of insulin resistance in skeletal muscle [61]. Increased levels of ROS within the cell due to insufficiency of intracellular antioxidants and disruption of the cellular redox state can lead to activation of serine/threonine kinases, such as JNK, p38 MAPK, and IKKβ/NF-κB [62]. Furthermore, high levels of ROS can cause damage to cellular organelles, especially mitochondria, through damage to DNA, proteins, and lipids, and disrupt their function, thereby causing insulin resistance. There is also evidence showing that ROS activates NF-κB, leading to increased inflammatory responses in skeletal muscle [63].

### 2.6 The link between endoplasmic reticulum and skeletal muscle insulin resistance

Endoplasmic reticulum (ER) stress can occur under conditions such as oxidative stress, inflammation, hyperglycemia, hyperlipidemia and hyperinsulinemia. ER stress plays a key role in the pathogenesis of insulin resistance in skeletal muscle by activating unfolded protein response (UPR)-related factors [39]. One of the factors involved in the UPR process during ER stress is the inositol-requiring enzyme 1α (IRE1α) protein. IRE1α, through its kinase domain, can activate the IKKB and JNK pathways resulting in serine phosphorylation of IRS-1 and then interfering with the insulin pathway in skeletal muscle [64]. Another factor involved in the UPR process is protein kinase RNA-like endoplasmic reticulum kinase (PERK), which increases the expression of pseudokinase tribbles 3 (TRB3), which can inhibit Akt and cause insulin resistance in skeletal muscle [65]. In addition, ER stress can induce inflammation due to the activation of the NF-kB pathway and therefore, indirectly play a role in the pathogenesis of insulin resistance in skeletal muscle [66].

### 2.7 the role of circadian clock disruption in muscle insulin resistance

Circadian clock disruption is another factor that has been reported to be linked to insulin resistance and type 2 diabetes [41]. Circadian clock misalignment in humans can be caused by factors such as shift work, social jet lag, sleep disorders, clock gene mutations, and exposure to artificial light-dark cycles [67]. Skeletal muscle, like other tissues throughout the body, has an autonomous molecular clock and generates circadian oscillations [68]. The transcription factors brain and muscle ARNT-Like 1 (BMAL1) and circadian locomotor output cycles kaput (CLOCK) and their target genes including period (PER), cryptochrome (CRY), retinoid-related orphan receptor (ROR), and nuclear receptor subfamily 1 group D (NR1D) are involved in regulation of the cellular circadian rhythm. The molecular clock in skeletal muscle has been shown to play a role in insulin sensitivity in this tissue by increasing GLUT4 translocation and also the effecting on histone deacetylase 3 (HDAC3) [69,70]. Animal and human studies have shown that the disruption of the molecular clock mechanism in skeletal muscle leads to obesity and insulin resistance [[71], [72], [73]].

## 3 The effects of resveratrol on cultured cells

Extensive in vitro studies have been conducted with the aim of investigating the effects of resveratrol on glucose metabolism and insulin resistance. Rat L6, mouse C2C12, and human primary muscle cells are the most commonly used cellular models to study skeletal muscle in vitro. This section reviews the main findings from in vitro studies on the effect of resveratrol on skeletal muscle cells (Table 1).

**Table 1:** In vitro studies on the effects of resveratrol on skeletal muscle insulin resistance.

Resveratrol dose	Model	Effects	Ref
**30 μmol/L**	C2C12	↑glucose uptake in a PI3K-dependent manner	[87]
**0.01**–**1 μM**	normal and insulin resistance-induced C2C12 myotubes	↑insulin-stimulated glucose uptake ↑phosphorylation of InsR, Akt, GSK-3β, and PDK ↑SIRT1 protein level ↓PTP1B protein and mRNA level	[77]
**trans-resveratrol, 100 μM**	C2C12	↑glucose uptake in the absence of insulin ↑AMPK and ACC phosphorylation ↑insulin sensitivity via AMPK activation (↑the effects of insulin in glucose uptake and Akt phosphorylation)	[90]
**100 μM**	L6 Myocyte	↑glucose uptake in the absence of insulin no change for Akt phosphorylation ↑AMPK phosphorylation ↓p-mTOR and p-p70 S6K no change for translocation of GLUT4	[82]
**0.1 μmol/l**	C2C12	↑glucose uptake and GLUT4 translocation through ER pathway ↑p-ER- increase p-P38, p-Erk, p-Akt, p-InsR ER-a-mediated	[100]
**50 μmol/l**	C2C12	↑AMPK activity and ACC phosphorylation ↑the NAD-to-NADH ratio	[81]
**1**–**100 μM**	L6 Myotubes	↑glucose uptake ↑GLUT4 translocation ↑p-AMPK and p-Akt	[89]
**5**–**100 μM**	normal and chronic hyperinsulinaemia and TNF-α induced insulin resistance L6, C2C12 myotubes and primary myotubes	↓IRS-1, IRS-2 Ser/Thr phosphorylation no change for IRS-1, IRS-2 Tyr phosphorylation ↓p-JNK ↑Akt Ser473 phosphorylation	[83]
**10**–**80 μM**	L6 skeletal muscle cells cytokine/LPS Treated	↓iNOS protein expression ↓Nitrite production ↑p-AMPK and p-ACC no change for Acetylated PGC-1α/PGC-1α	[86]
**0.1**–**200 μmol/l, 4h**	Primary human muscle cells Primary human muscle cells palmitate-induced insulin resistance L6	↓the insulin-stimulated glucose uptake ( ↓basal glucose uptake ↓basal and insulin-stimulated glycogen synthesis ↓basal and insulin-stimulated palmitate oxidation ↓insulin-stimulated p-AKT ( ↓p-AMPK and p-ACC ↑Acetylated PGC-1α/PGC-1α ( ↑ER stress (↑eIF2α phosphorylation, ↑CHOP levels) ↓basal and insulin-stimulated glycogen synthesis ↓insulin-stimulated p-AKT ↓p-AMPK and p-ACC ( ↑ER stress (↑eIF2α phosphorylation, ↑CHOP levels) ↓Insulin stimulated glucose uptake ( ↓basal glucose uptake (200 μmol/l) ↓insulin-stimulated p-AKT ↑p-AMPK and p-ACC ↑eIF2α phosphorylation and CHOP level ↑BiP levels	[153]
**10 μM**	C2C12 Myotubes	↑insulin-stimulated glucose uptake in an ERα dependent manner ↑insulin-stimulated GLUT4 translocation in an ERα- dependent manner ↑CAV-3 mRNA and protein expression –	[101]
**20 μM or 50 μM**	C2C12	↑p-AMPK and p-ACC ↑PGC-1α protein expression and activation ↑mitochondrial protein (COX I, COX IV, ATP SYNTHASE, CYT C, CS) dependent on AMPK and independent on SIRT1 pathway	[85]
**50 μM**	C2C12 and C2C12 overexpression of PGC-1 Primary mouse myoblasts	↓PGC-1α and UCP3 mRNA expression no change for PGC-1β, CytC, COX5B, CS, MCAD mRNA expression ↑PDK4 mRNA expression ↓p-AMPK no change for genes expression involved in either lipogenesis or cholesterol synthesis (SREBP1c, SCD1, FAS, ACC1, FDFT1, DHCR7) no change for PGC-1α, PGC-1β, CYT C, COX5B, CS, PDK4, UCP3, MCAD mRNA expression	[103]
**20, 60, 100 μM**	L6 myotubes	no change for basal glucose uptake ↓insulin-stimulated glucose uptake no change for effect on GLUT4 translocation and protein expression ↑SIRT1 protein expression ↑SIRT1activity no change for AMPK phosphorylation no change for acetylation status of FOXO1 ↑the production of NAD+ and ↑the NAD+/NADH ratio no change for mitochondrial mass ↑ATP production no change for ADP/ATP ratio ↑PGC-1α protein expression no change for NRF1 protein expression no change for the transcripts of the gene set of Mitochondrion organization and biogenesis and Insulin receptor signaling pathway in microarray analysis	[154]
**200 μM**	skeletal muscle from women treated with TNF-α, LPS, polyinosinic-polycytidylic acid (poly (I:C)) to induce a GDM-like model	↑insulin-stimulated phosphorylation of IR-β, IRS-1, GLUT4 protein ↑insulin-stimulated glucose uptake	[88]
**100 μM**	C2C12 palmitate-induced	↓TNF-α, IL-6 mRNA expression through a SIRT1-independent mechanism ↓IL-6 protein level through a SIRT1-independent mechanism ↓p-ERK1/2 and p-JNK and p-IKKα/IKKβ no change for p-P38 ↓ROS level (intracellular (O2-., H2O2) ↑NRf2 mRNA expression	[84]
**100 mM**	C2C12 palmitate-induced	↓senescence-associated β-galactosidase expression ↓autophagic flux ↓p16, p21 protein expression ↓LC3-II/LC3-I and P62 protein expression no change for p-AMPK and p-mTOR ↑p-AKT Ser473	[96]
**150 μmol/L**	L6 palmitate-induced	↓triglyceride content and cytoplasmic lipid droplets ↑protein expression of ATM, p-AMPK, COXIV, and CPT1 ↑glucose uptake	[91]
**50 μM**	C2C12 FFA-treated C2C12	↑insulin-stimulated p-Akt ↑glucose uptake alone no change for effect of insulin on glucose uptake ↓insulin-stimulated ROS and PIP3 generation ↑total GSH and GSH/GSSG ratio ↑insulin-induced AKT phosphorylation ↑glucose uptake with insulin co-treatment ↓FFA-induced ROS generation ↑total GSH and GSH/GSSG ratio ↑glucose uptake by change intracellular redox homeostasis	[97]
**, 10 μM**	L6 palmitate-induced	↑glycogen content ↑glucose uptake independent on CaMKII pathway but dependent on cPKC pathway ↑glucose uptake via scaffolding structure of actin cytoskeleton and microtubule ↑GLUT4 translocation utilizes non-TfR-trafficking ↑glucose uptake via cPKC and AMPK pathway but not PI3K/Akt, aPKC pathway	[92]
**25 μM**	L6 high insulin -induced	↑insulin-stimulated glucose uptake ↓Ser307 and Ser636/639 Phosphorylation of IRS-1 ↓p-mTOR/t-mTOR and p-p70 S6K ↑p-AMPK/t-AMPK ↑Insulin-Stimulated GLUT4 Translocation	[93]
**25 μM**	L6 palmitate-induced	↓Ser307 and Ser636/639 Phosphorylation of IRS-1 ↓p-mTOR/t-mTOR and p-p70 S6K ↑p-AMPK/t-AMPK and p-Akt/t-Akt ↑alone and Insulin-Stimulated glucose uptake ↑alone and Insulin-Stimulated GLUT4 Translocation	[94]
**20 μM**	C2C12 palmitate-induced	↓triglyceride content ↑glycogen content ↓ROS level	[98]
**10 μM**	Human primary skeletal muscle cells treated by siRNA depletion of OPA1	↓ROS level ↓NPAS2 (a paralog and substitute of Clock) expression	[99]
**Combination of FER (25 μM), resveratrol (10 μM) and EGCG (5 μM)**	Palmitate-treated L6 myotubes primary skeletal muscle cells	↑glucose uptake via GLUT4 ↑GLUT4 translocation ↑p-ACC protein and weakly p-Akt ↑glucose uptake complementarity of the endosome and GSVs trafficking in PI3K and AMPK signaling pathways ↓intramyocellular lipids ↑glucose uptake	[95]

ACC: Acetyl-CoA carboxylase, aPKC: atypical protein kinase C, ATM: ataxia telangiectasia mutated, BiP: Binding immunoglobulin protein, CaMKII: Ca2+/calmodulin-dependent protein kinase II, CAV-3: Caveolin 3, CHOP: CCAAT/enhancer-binding protein (C/EBP) homologous protein, COX: cytochrome oxidase, cPKC: Conventional protein kinase C, CPT-1: Carnitine palmitoyltransferase–1, CS: citrate synthase, Cyt: cytochrome, DHCR7: 7-dehydrocholesterol reductase, eIF2α: eukaryotic initiation factor 2α, ER: Estrogen receptor, ERK: extracellular signal-regulated kinase, FAS: fatty acid synthase, FDFT1: farnesyl-diphosphate farnesyltransferase 1, FOXO1: Forkhead box transcription factor O1, GSV: Glucose transporter storage vesicle, IKK: IκB kinase, InsR/IR: Insulin receptor, IRS-1: Insulin receptor substrate, JNK: c-Jun N-terminal Kinase, MCAD: medium-chain acyl-CoA dehydrogenase, NPAS2: Neuronal PAS domain protein 2, NRF-1: nuclear respiratory factor-1, PDK: pyruvate dehydrogenase kinase, PTP1B: Protein Tyrosine Phosphatase 1B, SCD1: stearoyl-CoA desaturase-1, SREBP: sterol regulatory element binding protein, TfR: transferrin receptor, UCP: uncoupling protein.

### 3.1 Activation of SIRT1 attenuates skeletal muscle insulin resistance

SIRT1, one of the seven members of the mammalian sirtuins family, is an NAD + -dependent deacetylase that plays a key role in insulin resistance in skeletal muscle. It has been shown that the reduction of SIRT1 expression is related to the development of insulin resistance, and increased its expression improves insulin sensitivity in skeletal muscle. The functional mechanism of SIRT1 in the insulin resistance of skeletal muscle is thought to be through the effect on improving mitochondrial dysfunction, insulin signaling, and reducing oxidative stress [74]. SIRT1 is involved in control of mitochondrial biogenesis and content through deacetylation of peroxisome proliferator-activated receptor γ coactivator 1-α (PGC-1α), a transcription factor coactivator [75]. Activation of PGC-1α by SIRT1 leads to activation of transcription factors related to the expression of mitochondrial biogenesis, lipid metabolism, FFAs oxidation, tricarboxylic acid cycle, and respiratory chain genes. PGC-1α also increases glucose uptake in skeletal muscle cells through increasing GLUT4 gene expression. In this way, PGC-1α is involved in improving insulin signaling as the reduction of PGC-1α in skeletal muscle cells was suggested to induce insulin resistance [76]. The direct effect of SIRT1 on the insulin signaling is through suppression of protein tyrosine phosphatase 1B (PTP1B) transcription, an enzyme that attenuates the insulin signaling pathway through dephosphorylation of INSR and IRS-1 [77].

### 3.2 Activation of AMPK attenuates skeletal muscle insulin resistance

AMP-activated protein kinase (AMPK) is another molecule that is linked to insulin resistance. AMPK is an αβγ heterotrimeric enzyme considered a key intracellular energy sensor and activated under the cell's low energy state (increasing the ratio of AMP: ATP). Muscle contraction (exercise), calorie restriction, AICAR, metformin, thiazolidinediones, leptin, and adiponectin are regarded as AMPK activators. Over the last two decades, the extensive studies have shown the importance of AMPK in skeletal muscle metabolism and its relationship with type 2 diabetes. Activated AMPK is thought to be a therapeutic target for type 2 diabetes and insulin resistance by stimulating glucose uptake, FFA oxidation, improving mitochondrial function, and inflammation in skeletal muscle. AMPK increases the translocation of GLUT4 to the membrane surface through phosphorylation of TBC1 domain family member 4 (TBC1D4). In addition, AMPK increases FFAs oxidation through acetyl-CoA carboxylase 2 (ACC2) phosphorylation. Also, AMPK activation directly by PGC-1α phosphorylation and indirectly by increasing NAD levels and SIRT1 activation improves mitochondrial function. Finally, AMPK activation has been shown to suppress inflammatory responses in skeletal muscle [78,79].

### 3.3 Resveratrol counteracts insulin resistance through activation of AMPK–SIRT1 pathways to enhance mitochondrial biogenesis, ameliorate inflammation and reduce oxidative stress

It has been demonstrated that resveratrol is considered as a SIRT1 and AMPK activator in myotubes [80,81], although there is some controversies. As the first studies performed, Sun et al. showed that resveratrol improves insulin sensitivity in C2C12 myotubes under normal and insulin resistance conditions by activating SIRT1 and suppressing PTP1B gene expression. They showed that SIRT1 suppresses PTP1B gene transcription at the chromatin level by deacetylating histone H3, which reduces the expression of PTP1B leading to improvement in the insulin signaling [77]. Similarly, Breen et al. showed that resveratrol increases glucose uptake in the absence of the insulin via SIRT1-dependent AMPK activation and not by insulin-dependent pathway. They suggested that this increase in glucose uptake may be due to an increase in GLUT4 intrinsic activity, but not an increase in GLUT4 translocation [82]. However, unlike previous studies, other researchers have shown different results. Frodo et al. reported that resveratrol reversed TNF-α-induced insulin resistance without affecting SIRT1. Resveratrol decreased the serine/threonine phosphorylation of IRS-1 and IRS-2 and increased the phosphorylation of Akt, through direct inhibition of protein kinases such as PI3K-mTOR and JNK, and not through activation of SIRT1, indicating that resveratrol may have multiple targets and exhibit pleiotropic effects [83]. Likewise, Sadeghi et al. showed that resveratrol improves inflammation induced by palmitate in C2C12 myotubes. In addition, resveratrol reduced the levels of ROS in skeletal muscle cells. Their results revealed that the mechanism involved in the effect of resveratrol on the improvement of inflammation in skeletal muscle cells is independent of SIRT1. The mechanism of the anti-inflammatory effect of resveratrol was due to its effect on pathways such as NF-kB and MAPK, as well as reducing the levels of ROS [84]. Moreover, Higashida et al. found that treatment with resveratrol increased mitochondrial proteins and induced AMPK activation in C2C12 myotubes, and this effect of resveratrol on mitochondrial biogenesis depends on the AMPK pathway rather than SIRT1. It is interesting to note that, in contrast to studies showing that resveratrol increases mitochondrial biogenesis by deacetylation PGC-1α through SIRT1 activation, they showed that deacetylation of PGC-1α by SIRT1 decreases PGC-1α activity and reduces mitochondrial protein expression. Therefore, they believe that the effects of resveratrol on mitochondrial biogenesis are due to increase in AMPK activity and, as a result, the activation of PGC-1α by AMPK [85]. The relation between the induction of inducible nitric oxide synthase (iNOS) in inflammation and the pathogenicity of insulin resistance in skeletal muscle has been determined. In this regard, Centeno-Baez et al. showed that resveratrol inhibits cytokines/lipopolysaccharides (LPS)-induced iNOS and nitrite production by the activation of AMPK but not SIRT1 [86].

A great deal of research has focused on the effects of resveratrol on AMPK and insulin signaling pathways. Resveratrol has been shown to enhance glucose uptake into C2C12 myotubes via a PI3K-dependent pathway in a dose-dependent manner [87]. Moreover, Tran et al. showed that resveratrol ameliorated insulin resistance in skeletal muscle tissue isolated from pregnant mothers treated with pro-inflammatory cytokines and LPS to induce a gestational diabetes-like model by improving insulin signaling [88]. Also, Minakawa et al. reported that resveratrol increases glucose uptake and translocation of GLUT4 through both AMPK and insulin signaling pathways [89]. However, Park et al. showed that *trans*-resveratrol increases glucose uptake in C2C12 cells in the absence of the insulin via AMPK activation and not by PI-3K/Akt pathway. They also showed that *trans*-resveratrol increases insulin sensitivity via AMPK activation because the stimulation of cells with insulin in the presence of resveratrol resulted in a significant increase in glucose uptake and Akt activity compared to the absence of resveratrol [90]. In a study, Zhang et al. showed that resveratrol decreased lipid content in palmitate-induced insulin-resistant L6 myotubes and improved insulin resistance. Ataxia-telangiectasia mutated (ATM) is one of the kinases that activate AMPK. They showed that resveratrol likely exerts this effect by activating the ATM-AMPK pathway, leading to improved β-oxidation and mitochondrial function [91]. Furthermore, Kang et al. showed that resveratrol ameliorates insulin resistance in a palmitate treated L6 cell model by facilitating GLUT4 storage vesicles (GSV) trafficking through an AMPK and PKC-dependent pathway rather than an insulin signaling-dependent pathway [92]. Likewise, Vlavcheski et al. showed that resveratrol improved insulin resistance induced by high insulin in L6 myotubes. They showed that resveratrol reduces the phosphorylation of the serine residue of IRS-1, which is probably phosphorylated by increasing the activity of mTOR and S6K. They suggested that, presumably, resveratrol exerts its effect on the inhibition of mTOR and S6K by activating AMPK and followed by reducing the phosphorylation of IRS-1 serine residue, increasing GLUT4 translocation and finally enhancing glucose uptake in skeletal muscle cells [93]. As well, Den Hartogh et al. showed that resveratrol improves FFA-induced insulin resistance in L6 myotubes. Their results showed that treatment of insulin-resistant cells with resveratrol decreases IRS-1 serine residue phosphorylation and increases Akt activity, GLUT4 translocation, and glucose uptake. They suggested that the mechanism of the action of resveratrol in improving insulin resistance may be due to activation of AMPK, which inhibits mTOR and S6K and thus inhibits IRS-1 serine phosphorylation [94]. In a study conducted by Bo Kang et al. , it was shown that combined treatment of resveratrol with ferulic acid and epigallocatechin gallate (EGCG) improved palmitate-induced insulin resistance in L6 myotubes through both effects on the insulin signaling and AMPK pathways [95]. Chang et al. showed that the impairment of autophagy flux in skeletal muscle is involved in the occurrence of cellular senescence and insulin resistance. They showed that resveratrol ameliorated cellular senescence and insulin resistance in C2C12 induced by palmitate through restoring autophagic flux. However, in this study, the insulin signaling pathway improved during resveratrol treatment, but this effect was independent of AMPK activation [96].

Resveratrol is able to improve insulin resistance by reducing oxidative stress. Quan et al. reported that resveratrol improves insulin sensitivity in palmitate-induced insulin resistance C2C12 myotubes by improving ROS homeostasis. They showed that resveratrol decreased insulin-stimulated Akt activity in C2C12 cells by inhibiting intracellular ROS generation [97]. In addition, Gong et al. showed that resveratrol improves lipid and glucose metabolism by reducing lipid accumulation and increasing glycogen storage in palmitate-treated C2C12 myotubes through reducing cellular oxidative stress [98]. Also, Gabriel et al. suggested that resveratrol decreased the level of ROS and attenuated impaired expression of core-clock genes caused by disrupted circadian oscillations in human primary skeletal muscle cells [99].

Recent studies have shown that Estradiol (E2) participates in glucose homeostasis through estrogen receptor (ER) signaling pathway. In one study, resveratrol was shown to improve glucose uptake in C2C12 cells in an ER pathway-dependent manner. In this study, resveratrol increased glucose uptake and translocation of GLUT4 through insulin-dependent and insulin-independent pathways. Furthermore, the results of this study demonstrated that the activation of both pathways by resveratrol is dependent on ER-α activation [100]. Also, Zhi Tan et al. reported that resveratrol increases the expression of CAV-3, one of the isoforms of caveolins, through ER-α, which leads to increase of GLUT4 translocation to the surface membrane, thereby improving glucose absorption [101].

Contrary to numerous studies demonstrating the beneficial effects of resveratrol on the improvement of insulin resistance in vitro, the results of several studies are contradictory. Moody et al. showed that resveratrol not only does not stimulate glucose uptake in L6 myotubes in the basal state, but also it has an inhibitory effect under insulin-stimulated conditions. Their results showed that the translocation of GLUT4 did not change during treatment with resveratrol, so resveratrol has no effect on improving insulin sensitivity. Also, their results showed that the protein expression of SIRT1 and PGC-1α were increased during treatment with resveratrol, but no significant change was seen in mitochondrial biogenesis [102]. Moreover, Asvenson et al. showed that resveratrol not only has no effect on the transcription of mitochondrial genes but also it reduces the expression of PGC-1α gene and AMPK protein [103]. In addition, Skrobuk et al. suggested that resveratrol treatment did not ameliorate palmitate-induced insulin resistance in human primary muscle cells. In this study, basal and insulin-stimulated glucose uptake were attenuated by resveratrol, and resveratrol had no effect on AMPK and SIRT-1 activation. Moreover, contrary to what was expected, resveratrol aggravated ER stress in primary and L6 myotubes [104]. The reason for the discrepancies between the studies remains unclear, however, some factors such as different cell line used, various concentrations and time of resveratrol treatments can be the potential reasons for the discrepancies between the studies.

Overall, the evidence from in vitro studies suggests that resveratrol can play an important role in improving glucose metabolism and protecting against insulin resistance in skeletal muscle cells, although some studies have shown controversial results. Data from these studies suggest that resveratrol treatment improves inflammation, oxidative stress, insulin signaling, and glucose uptake in normal and insulin-resistance skeletal muscle cells. Although the exact mechanism of its action in skeletal muscle is not clear, most studies have suggested the activation of AMPK by resveratrol. Furthermore, the SIRT1 and ER-α signaling pathway have also been demonstrated to play a role in resveratrol action.

## 4 Animal studies

In this section, we will review the animal studies that have investigated the effects of resveratrol on insulin resistance in skeletal muscle. This section includes studies on models of normal rodents, type 1 diabetics, and type 2 diabetics (Table 2).

**Table 2:** In vivo studies on the effects of resveratrol on skeletal muscle insulin resistance.

Resveratrol dose	Model	Effects	Ref
**200 or 400 mg/kg/day, 15WK**	male C57Bl/6J mice- HFD	↑mitochondrial size ↑mtDNA content ↑mitochondrial enzymatic activity (citrate synthase, succinate dehydrogenase) ↑oxidative capacity (↑maximum VO_2_ rate) ↑the ratio of oxidative to nonoxidative type-muscle fibers ↑PGC-1α protein- ↓ratio of PGC-1α deacetylated/acetylated (↑activity form) ↑PGC-1α, NRF-1, ERRα Tfam, UCP3 (Uncoupling ROS), Myoglobin (fiber type marker) mRNA expression	[123]
**0.5 mg/kg body, three times a day for 14 days**	STZ-induced diabetic rats	↑glucose uptake	[27]
**3 mg/kg, 3 times daily, Oral gavage 7 days**	STZ-induced diabetic male Wistar rats	↑p-Akt in a PI3K-dependent manner ↑GLUT4 protein level	[87]
**2.5 mg/kg/day**	mice fed a HFD	↑SIRT1 protein level ↓PTP-1B protein level	[77]
**(1 mg/kg. day), for either 15 days or 15 weeks**	Male Sprague-Dawley rats fed a high cholesterol–fructose (HCF)	↑glucose uptake ↑GLUT4 translocation ↑InsR phosphorylation ↑p-ER	[100]
**400 mg/kg/day, 12wk**	wild type C57BL/6J mice and AMPKa2 and AMPKa1 deficient mice fed a HFD	↑p-AMPK and p-ACC no change for SIRT1 level ↑both AMPKα1 and AMPKα2 activity ↑gene expression of PGC-1α, PGC-1β, MCAD only in wild-type mice but not in AMPK knockout mice ↑mitochondrial content (cytochrome C and mtDNA) only in wild-type mice but not in AMPK knockout mice ↓ROS levels in wild-type but not in AMPK knockout mice ↓DAG and ceramide levels only in wild-type mice but not in AMPK knockout mice ↑the NAD-to-NADH ratio and ↓PGC1 acetylation in wild-type but not in AMPK knockout mice (which may explain how resveratrol may activate Sirt1 indirectly)	[81]
**25 mg/kg, an ip injection during anesthesia**	Male Sprague Dawley rats	no change for p-Akt	[105]
**4 g/kg diet, 9wk**	Pregnant rats were exposed to hypoxic environment and then male offspring receive HFD	↓Triglyceride, DAG, ceramide levels ↑p-AMPK, p-ACC, p-Akt ↓p-IRS-1(Ser-1101 inhibitory) ↓p-PKCθ (active form)	[119]
**100 mg/kg/d, orally 11wk**	Yorkshire miniswine fed a high–fat/cholesterol diet	↑GLUT4, PGC-1α, p-Akt protein ↓RBP4 protein ↑PPAR-α and PPAR-γ protein level ↑GLUT4 translocation	[127]
**100 mg/kg body weight per day, orally 8wk**	Sprague Dawley Rat fed a HFD	↑glucose uptake ↓intramuscular FFA and Triglyceride ↑SS but not IMF mitochondrial citrate synthase and complex I to IV activity ↑CPT-1, MCAD, LCAD activity in SS mitochondrial ↑FAT/CD36, PGC-1α, NRF-1 and Tfam mRNA levels compared to HFD ↑SIRT1 activity	[115]
**0.75 mg/kg body wt, three times a day orally 8 wk**	Male Sprague-Dawley (SD) rats STZ induced	↑mtDNA ↓NF-kB protein level ↓IL-6, IL-1B mRNA expression	[114]
**40 mg/kg body weight, single intraperitoneal injection**	Male C57/BL6 mice intraperitoneal LPS	↓iNOS protein expression ↓NO production ↑p-AMPK and p-ACC	[86]
**100 mg/kg body weight per day, orally 12wk**	male Sprague-Dawley rats catch-up growth	↑glucose uptake ↑SIRT1 activity ↑PGC-1α, NRF-1, Tfam mRNA expression ↑mitochondrial number, volume density and citrate synthase activity in SS and IMF mitochondria ↑electron chain transport activity ↓mitochondrial ROS level ↑antioxidant enzyme activity include SOD, CAT, GPx	[124]
**30 mg/[kg d], oral gavage 2WK**	Male C57BL/6 N mice HFD	no change for p-Akt ↑p-AMPK no change for Glycogen content	[137]
**10 mg/kg/day, 16wk**	high-fat diet (HFD) ovariectomized rats	↑insulin-stimulated glucose uptake ↑insulin-stimulated GLUT4 translocation ↑CAV-3 mRNA and protein expression	[101]
**0.005 % and 0.02 %, w/w, 6WK**	C57BL/KsJ-db/db mice	↑GLUT4 and glycogen synthase protein expression ↑p-AMPK ↓p-ACC ↑UCP2, UCP3 and PPARα protein expression ↓p-IKKβ protein expression	[125]
**low dose (12.5 mg/kg diet) or high dose (225 mg/kg diet)**	C57BL/6 mice fed a HFD	no change for glucose uptake Resveratrol alone either at low or high doses ↑glucose uptake Low-dose resveratrol in combination with leucine and its metabolite HMB	[80]
**200 mg. kg−1 body weight, 6wk**	Male ZDF (Zucker diabetic fatty) rats	↑insulin-stimulated glucose uptake ↑Submaximal ADP-stimulated respiration no change for electron transport capacity ↑lipid-supported respiration rates no change for Mitochondrial content (complex I subunit, complex II subunit 30 kDa, complex III subunit core 2, complex IV subunit I, ATP synthase subunit alpha protein level) no change for ANT1 protein level ↑ANT2 protein level (ADP transport) ↓ H_2_O_2_ emission in the presence of ADP ↑ GSH/GSSG ratio	[128]
**4 g/kg food, 12 months**	Wild type (WT) and PGC-1α knockout (KO) healthy aging mice	no change for p-AMPK and SIRT1 no change for citrate synthase activity no change for mtDNA content no change for Oxidative protein content (PDH)-E1α and Cyt *c*-	[106]
**4 g per kg diet, 8 wk**	Male Wistar rats Male Wistar rats fed a HFD Male C57BL/6J mice fed a HFD	no change for PGC-1αprotein expression no change for mitochondrial enzyme proteins (NADH-UO, SUO, COX IV, CYT C, CS, LCAD)	[85]
**1, 10, and 100 μg/kg per day, oral intubation 1wk**	male Long-Evans rats STZ	↓superoxide anion levels in both muscle type (EDL & SOL) ↓protein expression of CuZnSOD in fast-twitch muscle (EDL) ↑protein expression of MnSOD in slow-twitch muscle (SOL) ↑p-Akt in slow muscle (SOL) no change for p-AMPK in both type muscle ↑GSK-3 phosphorylation in both type ↓p-ACC in slow muscle no change for p-mTOR	[113]
**20 mg/kg, i.p. 90 % transresveratrol, resVida, 8wk)**	Male Wistar albino rats STZ-induced	↑GLUT4, SIRT1 and visfatin (also known as PBEF or Nampt) protein level	[107]
**4 g/kg diet, 4 and 13 weeks**	control and PGC-1 muscle-specific knock-out (MKO) mice fed a HFD	↑PGC-1α mRNA level in HFD-control group ↑Cytc, Cox5b, and Cs mRNA expression (PGC-1 target genes) in HFD-control group but not in MKO group no change for OXPHOS protein (mitochondrial protein) levels no change for oxidative enzymatic staining no change for UCP3 mRNA expression no change for fatty acid B-oxidation genes (Mcad, Cpt1b, Pdk4) expression no change for GLUT4, HK2, PKM1 mRNA expression no change for p-AMPK	[103]
**(3 mg/kg/h, intravenous)**	female Wistar rats Intralipid plus heparin intravenous	↓p-IRS-1 Ser307 ↑total IRS-1 and p-Akt protein ↓phosphorylation of IkBα ↑total IkBα no change for Total and phosphorylated levels of mTOR, p70 S6 kinase, JNK, AMPK ↑MDA level	[134]
**2.5 mg/kg/day, orally 8 weeks**	Irs2-deficient mice	↑INSR (Tyr) and Akt insulin-induced phosphorylation ↓PTP1B mRNA expression	[129]
**100 mg/kg body weight/day, intragastric administration 8WK**	Male SD (Sprague Dawley) rats fed a HFD	↑glucose uptake ↑p-Akt ↑SIRT1 protein lelvel and activity ↑SIRT3 mRNA and protein expression ↑mtDNA copy number ↑PGC-1α mRNA and protein level ↑NRF-1 and Tfam gene expression ↓ROS and MDA level in both SS and IMF mitochondria ↑SOD, CAT and GPx activities in both SS and IMF mitochondria	[155]
**10 mg/kg body weight and Low-fat diet, orally gavaged, once per day, 5 day/week for 8 weeks**	Male C57BL/6 mice fed a HFD	no change for TNF-α, IL-6, MCP-1 gene expression no change for CPT-1 and ACOX, SREBP, ACC, FAS gene expression	[139]
**100 mg/kg/day. 4WK**	C57BL6 mice fed a HFD	no change for p-Akt (Ser) alone but ↑p-Akt with combination with metformin under insulin-stimulated	[138]
**25 mg/kg/d, 36 days**	C57BL/6 kept in constant darkness for induction insulin resistance	↑SIRT1 protein ↑p-Akt, p-INSR and p-GS3Kβ	[136]
**50 mg/kg, orally**	ICR male mice fed a HFD	↓DAG contents ↓PKCθ translocation ↑p-Akt insulin-mediated	[120]
**100 mg/kg body weight/day, intraperitoneal injection 8 weeks**	Male C57/BL6 mice fed a HFD	↓Triglyceride content no change for glycogen content ↑myosin I and IIx mRNA expression ↓myosin IIb mRNA expression ↑Cidea mRNA expression no change for mitophagy and autophagy gene expression	[116]
**30 mg/kg body weight/day, 6 weeks**	male Wistar rats fed a high-fat high-sucrose (HFHS) diet	no change for Triglyceridecontenet no change for ratio p-InsRβ (Tyr1162-1163)/total InsRβ, p-Akt and p-AS160, GLUT4	[140]
**30 mg/kg of body weight, 60 days**	Type 2 diabetic CD1 mice induced by neonatal injection of monosodium glutamate	↑Slc2a4 mRNA level ↑GLUT4 protein ↑SIRT1 nuclear content	[130]
**0.1, 1, 10 mg/kg, subcutaneous injection 4wk**	female ovariectomized Kunming mice STZ -induced	↑GLUT4 mRNA level ↑GLUT4, IRS-1 protein level ↓p-ERK protein level	[112]
**50, 100 or 200 mg/kg body weight, 22 days**	Male Wistar rats fed a cafeteria diet	↓total lipid content no change for Cpt1b, Pgc1a, Ucp2 and Ucp3 gene expression ↑leptin sensivity index (pSTAT3/serum concentration of Leptin) no change for iNOS mRNA expression no change for transcripts related to ER stress ↓SIRT1 activity ↑Leptin Receptor (ObRb)	[141]
**1, 5, and 10 mg/kg body weight/day, gavage 30 day**	male Wistar rats STZ-NA induce type 2 diabetes	↓SNAP-23, syntaxin-4, and VAMP-2 mRNA expression	[132]
**20 mg/kg b.w. gavage 10wk**	male Goto-Kakizaki (GK) rats	↓TG content no change for NEFA significantly no change for AMPK and p-AMPK protein ↑ p-ACC, Akt and P-Akt protein	[121]
**100 mg/kg feed/d, 8 wk**	C57BL6/J male mice fed a HFD	↓TG content and lipid accumulation ↑protein expression of ATM, p-AMPK, COXIV, and CPT1	[91]
**30 mg/kg of body weight, 2 months**	neonatal subcutaneous injections of monosodium glutamate (MSG) in male offspring of CD1 mouse	↑Slc2a4 mRNA and GLUT4 protein no change for H3Kac content ↓H3K9me3 content in the Slc2a4 segment ↑MEF2A/D binding activity on enhancer segment of Slc2a4 ↑p-AMPK ↑SIRT1 both nuclear and cytosolic protein content	[131]
**20 mg/kg intraperitoneally 20 days**	Male C57BL/6J mice fed a HFD	↑Insulin-induced glucose uptake ↓ROS level ↑total GSH and GSH/GSSG ratio	[97]
**20 mg/kg Body weight, 10wk**	Goto-Kakizaki (GK) rats	↓IL-6, TNF-α protein level no change for IL-1βB, NF-kB protein level ↓MDA level	[135]
**0.4 %, 16wk**	male C57/BL6 mice fed a HFD	↓CD11b and F4/80 marker ↓F4/80a and CD11b mRNA level ↓CD11c and increase CD206 marker ↓iNOS and CD11c mRNA level ↑CD206 and Arginase mRNA level no change for CD3 marker ↓CD8 and ↑CD4 and FOXP3 ↓TLR2, TLR4, TNF-α, IL-6, IL-1B, MCP-1 mRNA level ↑IL-10 mRNA level N = no change for RANTES mRNA level ↓ TNF-α and IL-6 protein level ↓p-P38, p-JNK, p65(NF-kB) ↑p-AMPK ↓lipid content	[156]
**15 mg/kg, intraperitoneally injected 10wk**	C57BL/6J mice fed a HFD	↓TG content ↑glycogen content ↓ROS level	[98]
**260 mg/kg, orally once a day 4 wk**	male Sprague-Dawley rats fed a HFD	↑p-AMPK/AMPK ↑SIRT1 and PGC-1α mRNA and protein level	[157]
**20 mg/kg b.w. gavage once a day 10 wk**	male Goto-Kakizaki (GK) rats	↑IR and p-IR protein level ↑insulin binding to IR no change for GLUT4 protein level ↑TUG protein level	[133]
**Neonatal treatment, 2 mg/kg body weight, from day 2 to 20 of age**	NMRI mice fed a HFD	↓TG and intramyocellular neutral lipid droplets ↑fatty acid oxidation/lipolysis gene expression (cpt1b, ucp3, ucp2, pnpla2) ↑mitochondria biogenesis and function gene expression (tfam, gabpa) no change for Tfb2m, Nrf1, Ppargc1a and Ppargc1b gene expression ↑prkaa2 (encode AMPK catalytic subunit isoform 2), sirt1and Gadd45a (target gene of the SIRT1) gene expression ↑p-AMPK protein level	[122]
**30 mg/kg body wt, intraperitoneal injection every other day 4 wk**	BALB/c white albino male mice fed a HFD	↑SIRT1, SIRT3, PGC-1α mRNA expression no change for AMPK gene expression no change for LCAD, β-HAD gene expression ↑LCAD, β-HAD activity ↓TG. DAG and ceramide content	[117]

ACC: Acetyl-CoA carboxylase, ACOX: acyl-CoA oxidase, ANT: adenine nucleotide translocase, ATM: ataxia telangiectasia mutated, CAT: catalase, CAV-3: CAV-3, Cidea: cell death-inducing DFFA-like effector A, COX: cytochrome oxidase, CPT-1: Carnitine palmitoyltransferase–1, CS: citrate synthase, Cyt: cytochrome, EDL: extensor digitorum longus, ER: Estrogen receptor, ERK: extracellular signal-regulated kinase, ERRα: estrogen-related receptor α, FAS: fatty acid synthase, FAT/CD36: fatty acid transport protein, FOXP3: forkhead box protein 3, GPx: glutathione peroxidase, GSH/GSSG: Glutathione/oxidized glutathione, H3K9me3: tri-methylation at lysine 9 of histone 3, HK2: hexokinase 2, HMB: β-hydroxy-β-methyl butyrate, IKKβ: I kappa B kinase, IMF: Intermyofibrillar, InsR/IR: Insulin receptor, LCAD: long-chain acyl-CoA dehydrogenase, MCAD: medium-chain acyl-CoA dehydrogenase, MDA: Malondialdehyde, MEF2A/D: myocyte-specific enhancer factor 2 A and D, NADH-UO: NADH ubiquinol oxidoreductase, Nampt: Nicotinamide phosphoribosyl transferase, NEFA: Non-esterified fatty acids, NRF-1: nuclear respiratory factor-1, PBEF: pre-Bcell colony-enhancing factor, PDH: pyruvate dehydrogenase, PDK4: pyruvate dehydrogenase kinase isozyme 4, PKM1: pyruvate kinase muscle isozyme 1, PNPLA2: patatin-like phospholipase domain-containing 2, RANTES: regulated on activation normal T cell expressed and secreted, RBP4: retinol binding protein 4, Slc2a4: Solute carrier family-2-member-4, SNAP: Synaptosome associated protein, SOD: superoxide dismutase, SOL: soleus, SREBP: sterol regulatory element binding protein, SS: Subsarcolemmal, STAT: signal transducer and activator of transcription, SUO: succinate ubiquinol oxidoreductase, Tfam: Mitochondrial transcription factor A, TFB2M: Transcription Factor B2 of the Mitochondria, TUG: Tether containing a UBX domain for GLUT4, UCP: uncoupling protein, VAMP-2: Vesicle associated membrane protein 2, β-HAD: β-Hydroxyacyl CoA dehydrogenase.

### 4.1 Studies on normal models

Studies investigating the effects of resveratrol on skeletal muscle insulin sensitivity in normal models are limited. A study showed that acute intraperitoneal injection of resveratrol could not increase Akt phosphorylation [105]. Furthermore, Ringholm et al. demonstrated that not only the lifelong consumption of resveratrol (for 12 months from 3 months of age) alone had no effect on improving the biogenesis and oxidative capacity of skeletal muscle mitochondria in aging mice, but it also failed to enhance the effects of lifelong exercise. Moreover, their findings showed that resveratrol could not increase the activity of AMPK and SIRT1 [106]. Further, in another study, resveratrol had no effect on the expression of SIRT1 and GLUT4 proteins in skeletal muscle of rats [107]. Other studies also found that resveratrol did not have beneficial metabolic effects in normal models [[108], [109], [110], [111]]. Since the studies conducted on skeletal muscle in normal models are limited, more studies are required to further clarify the effects of resveratrol on factors involved in insulin sensitivity in skeletal muscle in normal non-diabetic animal models.

### 4.2 Studies on type 1 diabetic models

Studies on type 1 diabetic rodent model show the beneficial effect of resveratrol on improving insulin resistance in skeletal muscle. In streptozotocin (STZ)-induced diabetic rats, treatment with resveratrol increased GLUT4 expression and glucose uptake in skeletal muscle [27,87,107,112]. The mechanism of resveratrol is probably through improving PI3K/Akt [87,113] and inhibiting MAPK/ERK [112] signaling pathways independently of AMPK activation [113]. Furthermore, Chen et al. showed that resveratrol reduced inflammatory markers in skeletal muscle of STZ-induced diabetic rats. Also, their metabolomics results showed that according to urinary and serum metabolites, as well as increased mitochondrial content in skeletal muscle, resveratrol might improve mitochondrial function and lipid metabolism disturbance in the state of diabetic conditions [114]. In addition, Chang et al. showed that resveratrol reduced oxidative stress in both slow-twitch and fast-twitch muscle fibers in STZ-induced diabetic rats. However, the role of resveratrol in regulating antioxidant enzymes was fiber-type-specific [113]. Therefore, resveratrol, with underlying mechanisms, including improving the insulin signaling pathway, mitochondrial function, reducing inflammation, and oxidative stress, can improve insulin resistance in the skeletal muscle of type 1 diabetic rodents.

### 4.3 Studies on type 2 diabetic models

#### 4.3.1 Resveratrolameliorates insulin resistance through reducing lipid accumulation in skeletal muscle

IMCL and lipid metabolites accumulation is associated with insulin resistance in skeletal muscle, as discussed earlier. Several studies have shown the effect of resveratrol on reducing lipid accumulation in skeletal muscle and subsequently improving insulin resistance in animal models [81,91,98,[115], [116], [117], [118]]. Dolinsky et al. suggested that resveratrol could improve HFD-induced insulin resistance in skeletal muscle of rats born to intrauterine growth restriction (IUGR) pregnant mothers. Resveratrol protected the reduction of IRS-1 and Akt activity by decreasing lipid metabolites and the active form of PKCθ [119]. Also, Zhao et al. showed that resveratrol improves insulin signaling by reducing lipid metabolites and activating PKCθ in skeletal muscle. They believed that resveratrol improves insulin resistance in skeletal muscle by reducing lipolysis of adipose tissue, thereby reducing FFAs flux into skeletal muscle [120]. In another study, Szkudelska et al. reported that resveratrol reduces lipid content in skeletal muscle of non-obese type 2 diabetic rats and improves insulin resistance. They showed that the effect of resveratrol on reducing muscle lipid content is independent of AMPK pathway [121]. In a study on mice, Serrano et al. demonstrated that resveratrol supplementation in the early days of infancy causes long-term beneficial effects on lipid metabolism in the later stages of their lives [122].

#### 4.3.2 Resveratrol ameliorates insulin resistance through improving mitochondrial function in skeletal muscle

Many studies have shown that mitochondrial function in skeletal muscle is improved under resveratrol treatment in type 2 diabetic models. Resveratrol increased oxidative and aerobic capacity, activity of subsarcolemmal and intermyofibrillar mitochondrial electron transport chain (ETC) complexes, biogenesis and the number and the density of mitochondria, myofiber remodeling, and lipid oxidation in skeletal muscle [81,91,115,117,119,[122], [123], [124], [125], [126]]. Also, resveratrol increased the expression levels of peroxisome proliferator-activated receptor α (PPAR-α), a transcription factor involved in fatty acid metabolism and metabolic homeostasis [127]. As mentioned earlier, mitochondrial dysfunction is related to lipid metabolites accumulation, increase in ROS levels, and ultimately insulin resistance in skeletal muscle. In a study on zucker diabetic fatty (ZDF) diabetic rats, Smith et al. found the relationship between ROS production and mitochondrial function with insulin resistance in skeletal muscle. In addition, they found that resveratrol can increase insulin sensitivity in skeletal muscles by enhancing mitochondrial function and decreasing ROS production [128]. The mechanism of the action of resveratrol on improving mitochondrial function in skeletal muscle is unclear, however, several studies have noted its molecular mechanism via activating SIRT1/PGC-1α [103,115,117,123,124,126] and some through the AMPK pathway [91,119,125].

#### 4.3.3 Resveratrol inhibits insulin resistance through promoting GLUT4 translocation in skeletal muscle

Several studies have shown improved insulin signaling and enhanced GLUT4 translocation following resveratrol treatment in animal models of insulin resistance. Sun et al. reported that resveratrol improved insulin resistance in skeletal muscle by affecting SIRT1 and suppressing PTP1B gene expression [77]. In another study on Irs2-deficient (Irs2−/−) mice, González-Rodríguez et al. showed that resveratrol reduced the expression of PTP1B gene and improved insulin resistance in skeletal muscle. However, since the use of overexpressing Sirt1 (*Irs2*−/−*Sirt1tg*) mice in this study did not play a role in this improvement, they did not consider the mechanism of resveratrol's effect on improving insulin sensitivity to be directly related to SIRT1 [129]. In addition, Deng et al. showed that resveratrol improves insulin resistance in skeletal muscle in an estrogen receptor (ER) pathway-dependent manner [100]. Similarly, Zhi Tan et al. showed that resveratrol increases the expression of CAV-3, possibly through an effect on the ER, thereby increasing the translocation of GLUT4 to the plasma membrane and thus improving glucose uptake and insulin resistance in skeletal muscle [101]. Yonamine et al. showed that resveratrol improves glucose homeostasis in type 2 diabetic rats. They demonstrated that GLUT4 gene and protein expression were increased in skeletal muscle during resveratrol treatment and suggested that SIRT1 may play a role in this regulation of gene expression [130]. Also, in another study, they showed that epigenetic modifications play a role in insulin resistance through decreasing the expression of Solute carrier family-2-member-4 (Slc2a4) gene. They suggested that AMPK and SIRT1 activation by resveratrol seems to play a role in this process [131]. Moreover, Farimani et al. showed that in the skeletal muscle of type 2 diabetic mice, the expression of Soluble N-ethylmaleimide-sensitive factor attachment protein receptor (SNARE) complex genes, which is involved in the process of translocation of GLUT4 to the membrane, is increased. They showed that resveratrol reversed the increased expression of synaptosome-associated protein 23 (SNAP-23), syntaxin-4, and vesicle-associated membrane protein 2 (VAMP-2) genes in skeletal muscle tissue [132]. In a study on Goto-Kakizaki (GK) rats, Szkudelska et al. found that resveratrol could increase the capacity and activity of the insulin receptor and its insulin binding in the skeletal muscle. They also showed that tether, containing a UBX domain, for GLUT4 (TUG) an important factor in GLUT4 translocation, was increased by resveratrol [133]. Also, GM Do et al. showed that resveratrol increased GLUT4 expression and glycogen synthesis through increasing AMPK activity [125]. On the other hand, Burgess et al. demonstrated that resveratrol improved insulin sensitivity in the skeletal muscle of metabolic syndrome swine model with increased GLUT4 translocation through reducing the level of retinol binding protein 4 (RBP4), and increasing PGC-1α [127].

#### 4.3.4 Resveratrol attenuates insulin resistance through inhibiting inflammation and oxidative stress in skeletal muscle

Chronic inflammation and oxidative stress, as discussed earlier, are other factors associated with insulin resistance in skeletal muscle. It has been demonstrated that resveratrol improves oxidative stress and ROS levels in skeletal muscle of type 2 diabetic models [97,98,128]. Um et al. showed that resveratrol decreased the levels of ROS in skeletal muscle through the AMPK-dependent pathway [81]. Similarly, Zheng et al. demonstrated that resveratrol decreased ROS levels induced by refeeding after caloric restriction, known as catch-up growth (CUG), and increased the activity of antioxidant enzymes [124]. In a study on rats fed a HFD, Haohao et al. found that resveratrol improves insulin resistance in skeletal muscle. Their results showed that oxidative stress and antioxidant enzyme activity were improved in both subsarcolemmal and intermyofibrillar mitochondrial populations. They found that resveratrol normalizes the imbalance between oxidative stress and antioxidant response caused by HFD in the subsarcolemmal mitochondria of skeletal muscle, and this may be due to the increased activity of SIRT1/PGC-1α and its effect on SIRT3 [126]. In addition, Pereira et al. showed that resveratrol improves insulin resistance caused by short-term lipid infusion in peripheral tissues. In this study, resveratrol reversed the adverse effects of FFAs on insulin signaling. Furthermore, the level of phosphorylation of IκBα, which was increased by lipid infusion, was reduced by resveratrol, and they suggested that the functional mechanism of resveratrol in improving insulin signaling is probably due to the reduction of IKKB, which phosphorylates IRS-1 at the serine residue, while the level of other protein kinases related to the insulin signaling such as JNK, mTOR, and AMPK did not change [134]. Similarly, Do et al. showed that resveratrol, by reducing the active form of IKKB, can play a role in relieving inflammation and improving the insulin signaling pathway [125]. Also, Szkudelska et al. showed that in non-obese GK rats model, resveratrol improved skeletal muscle inflammation and oxidative stress compared to the control group, and since resveratrol had no effect on the NF-κB pathway, it seems that resveratrol exerts its anti-inflammatory effects independently of NF-κB [135]. Furthermore, Shabani et al. showed that resveratrol has an anti-inflammatory impact on the skeletal muscle of mice fed an HFD. Their results demonstrated that the consumption of resveratrol for 16 weeks decreased the infiltration of macrophages into the skeletal muscle and increased the polarization of macrophages to M2 anti-inflammatory phenotype. Also, in addition to macrophages, resveratrol had a positive effect on T cells in skeletal muscle, so that CD8^+^ T cells were decreased and Tregs were increased. The results of measuring proinflammatory cytokines and signaling pathways involved also showed a reduction of inflammation in skeletal muscle. In addition, lipid content was also decreased in skeletal muscle during treatment with resveratrol. Their results also showed that AMPK activity was increased during resveratrol treatment, and they suggested that the anti-inflammatory effects of resveratrol may be exerted through activation of the AMPK pathway [118].

#### 4.3.5 Resveratrol attenuates insulin resistance through inhibiting circadian rhythm disruption in skeletal muscle

Finally, one study shows the beneficial effect of resveratrol on the improvement of insulin resistance induced by circadian rhythm disruption in skeletal muscle. Jun Liu et al. showed that insulin resistance caused by circadian clock misalignment is improved by resveratrol. They showed that resveratrol increases the level of SIRT1, which is appeared in constant dark conditions under the influence of the downregulation of CLOCK and BMAL1 transcription factors, thereby increasing insulin sensitivity in skeletal muscle [136].

#### 4.3.6 Contradictory findings on the effects of resveratrol on skeletal muscle insulin resistance

In contrast to studies demonstrating the beneficial effects of resveratrol on insulin resistance in animal models, the findings of a number of studies are the contradictory. Kang et al. showed that despite the AMPK activation, resveratrol had no impact on improving insulin signaling and increasing glycogen content in skeletal muscle [137]. Also, Brookbauer et al. found that resveratrol treatment failed to improve muscle glucose uptake in mice fed a HFD [80]. Similarly, Frendo‐Cumbo et al. showed that resveratrol alone had no effect on improving insulin resistance and could not increase Akt activity. However, the combined treatment of resveratrol with metformin could improve whole-body glucose homeostasis compared to individual treatment [138]. Moreover, Higashida et al. found that resveratrol supplementation had no influence on mitochondrial biogenesis in the skeletal muscle of mice and rats fed a normal diet and those fed a HFD [85]. Furthermore, Svensson et al. indicated that although resveratrol increased the transcription of genes involved in mitochondrial biogenesis in skeletal muscle in a PGC-1α-dependent manner, but it had no effect on the expression of mitochondrial oxidative proteins. They also showed that resveratrol had no effect on AMPK activation and upregulation of genes involved in fatty acid oxidation and glycolysis [103]. Also, Jeong et al. study in mice fed a HFD showed that consumption of resveratrol had no effect on inflammatory markers and lipid metabolism in skeletal muscle compared to mice fed a low-fat diet alone [139]. Additionally, Qi et al. demonstrated that resveratrol reduced the accumulation of triglycerides in the skeletal muscle of mice fed HFD. However, it could not compensate for the suppression of genes involved in mitophagy and autophagy due to HFD intake. Also, resveratrol had no effect on glycogen contents and fatty acid oxidation in skeletal muscle [116]. Similarly, Milton-Laskibar et al. indicated that resveratrol treatment with a standard diet had no effect on reducing lipid content and improving insulin signaling in skeletal muscle of rats with insulin resistance caused by an obesity diet [140]. Besides, Ardid-Ruiz et al. showed that resveratrol does not change the expression of genes related to fatty acid oxidation, mitochondrial biogenesis, thermogenesis, ER stress, and inflammation in the skeletal muscle of rats fed an obesity diet. Nevertheless, the sensitivity to leptin in skeletal muscle was significantly increased, which was parallel to a decrease in the lipid content of skeletal muscle. Furthermore, contrary to the expectations, SIRT1 activity was decreased in muscle, and they concluded that increased sensitivity to leptin in skeletal muscle tissue is independent of SIRT1 [141].

Collectively, most studies on models of type 2 diabetics show that resveratrol can be effective in improving insulin resistance in skeletal muscle and consequently, the whole body through reducing the accumulation of lipid metabolites, improving insulin signaling, mitochondrial function, lipid and glucose metabolism, reducing inflammation and oxidative stress in skeletal muscle. The exact functional mechanism of resveratrol needs to be clarified; however, it seems that resveratrol has pleiotropic effects on skeletal muscle and works through different mechanisms under various conditions. Nonetheless, according to the results of the most studies, AMPK and SIRT1 pathways seem to be the key mechanisms of resveratrol action.

## 5 Human studies

Over the last decade, a number of human studies have investigated the impact of resveratrol on insulin sensitivity in skeletal muscle. However, in contrast to animal studies, the results of the effects of resveratrol on parameters related to insulin resistance in skeletal muscle are inconsistent in human clinical trial studies (Table 3).

**Table 3:** Human studies on the effects of resveratrol on skeletal muscle insulin resistance.

Resveratrol dose	Model	Effects	Ref
**trans-RSV (resVida™) 150 mg/day, 30 days**	Randomized, placebo-controlled, double-blinded crossover study. 11 Healthy, obese men subjects	↑Muscle mitochondrial respiration ↑p-AMPK, SIRT1 and PGC-1α protein levels ↑Intramyocellular lipid levels ↑increase citrate synthase activity ↑oxidative phosphorylation-related gene expression ↓inflammation pathway and cytokine signaling-related gene expression no change for mitochondrial content no change for mitochondrial density	[142]
**trans-RSV (resVida™) 75 mg/day, 12 wk**	randomized, double-blind, placebo-controlled trial, non-obese, postmenopausal women with normal glucose tolerance (n = 45)	no change for skeletal muscle insulin sensitivity in two-stage hyperinsulinemic euglycemic clamp no change for SIRT1, NAMPT, PGC1α, UCP-3 mRNA expression no change for AMPK phosphorylation	[145]
**tablets containing 500 mg trans-RSV, 4 wk**	Double blinded, randomized placebo-controlled, parallel-group trial, 24 healthy obese male	no change for intramyocellular lipid content no change for p-AMPK and p-ACC no change for SIRT1-mediated deacetylase activity ↓GLUT4 mRNA expression no change for PGC-1α mRNA expression	[146]
**Trans-RSV, 3 g/d, 12 wk**	randomized, double-blind, parallel group, 10 subjects with T2DM	↑SIRT1 protein expression ↑p-AMPK/AMPK no change for PGC-1α protein expression no change for GLUT4 protein expression significantly no change for muscle type switching	[143]
**RSV, 250 mg, daily intake 8 wk**	43 aged human subjects. Healthy, physically inactive men	no change for AMPK, SIRT1 and ACC phosphorylation ↓total acetylation of lysine residues no change for PGC-1α mRNA expression no change for citrate synthase and 3-hydroxyacyl-CoA dehydrogenase activity no change for cyt C and COXI protein content no change for protein carbonylation level no change for IkB-α and IkB-β protein no change for JNK, p65, p38 and IKK phosphorylation no change for TNF-α, iNOS mRNA and protein expression	[147]
**combination of** **EGCG and trans-RSV supplements, 282 and 80 mg/d, respectively, 12wk**	randomized, double-blind, placebo-controlled, parallel intervention trial 38 overweight and obese subjects	↑basal respiration mitochondria (state 3) by ADP (malate and glutamate as substrates) no change for ADP-stimulated complex I–linked respiration (malate + glutamat) no change for respiration with a fatty acid substrate (malate + octanoylcarnitine) ↑the electron input of both complexes I and II ↑maximal mitochondrial respiration no change for Mitochondrial proton leak ↑complexes III and IV OxPhos protein content ↑citric acid cycle and respiratory electron transport chain transcriptional microarray no change for total lipid content or saturation of the triacylglycerol and diacylglycerol fractions	[152]
**trans-RSV (resVida™) 150 mg/day, 30 days**	17 well-controlled subjects with type 2 diabetes (T2D) a randomized double-blind crossover study	↑Intramyocellular lipid content ↑ADP-stimulated respiration no change for state 4 respiration upon addition of oligomycin no change for mitochondrial DNA content no change for mitochondrial mean protein content of the OXPHOS complexes no change for p-AMPK and PGC-1α protein content	[148]
**Trans-RSV, 1000 mg/d (high-dose RSV) and 150 mg/d (low-dose RSV), 16wk**	randomized, double-blind, placebo controlled parallel group clinical trial in male volunteers with the metabolic syndrome,74	no change for intramyocellular lipid no change for gene expression of TNF-α, IL-6, IGF-1, IGF-2, SIRT1, NRF1, or TFAM no change for phosphorylation of AMPK and ACC no change for total acetylation status of lysine residues	[149]
**RSV, 1g (500 mg, twice daily), 4 month**	45 middle-aged men with metabolic syndrome, a randomized, placebo-controlled, double-blinded, single-center study	↓intracellular lipids	[144]
**RSV, 2−3 g/daily, 6wk**	randomized, double-blind crossover study, older glucose-intolerant adults (n = 30)	↑mitochondrial number no change for mitochondrial size and morphology mitochondrial dysfunction and oxidative phosphorylation pathways were perturbed in RNA-Seq analysis ↓IL-2 expression, IL-1 signaling and SAPK/JNK signaling in RNA-Seq analysis	[150]
**Trans-RSV, 150 mg/day and placebo, 30 days**	randomized, placebo controlled, cross-over trial, Thirteen overweight male first degree relatives (FDR) of patients with T2D	no change for IMCL in neither type I or type II muscle fibers ↑mitochondrial ADP-stimulated respiration no change for Maximal FCCP-induced uncoupled respiration (sate u) significantly no change for protein content of individual OXPHOS complexes no change for mitochondrial PCr recovery half-time	[151]

ACC: Acetyl-CoA carboxylase, COXI: cytochrome *c* oxidase I, cyt: cytochrome, FCCP: Carbonyl cyanide p-(trifluoromethyl)-phenylhydrazone, IGF: insulin-like growth factor, IκB-α: inhibitor of κB-α, IκB-β: inhibitor of κB-β, NAMPT: Nicotinamide phosphoribosyltransferase, NRF1: nuclear factor erythroid-derived 2-related factor 1, OxPhos: oxidative phosphorylation, PCr: phosphocreatine, RSV: Resveratrol, SAPK: Stress-activated protein kinase, TFAM: Mitochondrial transcription factor A, UCP-3, Uncoupling protein 3.

In a study on obese subjects, Timmers et al. indicated that resveratrol has a similar effect to calorie restriction in the body's metabolism. In this study, mitochondrial activity, fat oxidative capacity, and mitochondrial respiration rate were increased in skeletal muscle tissue as a result of activation of AMPK and, subsequently, SIRT1 and PGC1-α activation [142]. Also, Goh et al. showed that resveratrol might induce exercise-mimicking effects by activating AMPK and SIRT1 in the skeletal muscle of subjects with type 2 diabetes because the resting metabolic rate has increased despite a decrease in daily activity [143]. In another study of middle-aged men with metabolic syndrome, Korsholm et al. reported that resveratrol changes pathways related to lipid and fatty acid metabolism and decreased intracellular lipid levels in skeletal muscle [144].

In contrast to previous studies, Yoshino et al., in a 12-week study on non-obese, postmenopausal women without impaired glucose tolerance, showed that resveratrol had no effect on the parameters evaluated. They suggested that insulin sensitivity in skeletal muscle did not change during resveratrol treatment, and the expression of factors involved in the AMPK pathway, mitochondrial function, and inflammation, which are targets of resveratrol, remained unchanged [145]. In a clinical study on 24 healthy obese subjects with mild insulin resistance, Poulsen et al. demonstrated that four weeks of resveratrol supplementation had no effect on improving insulin resistance, glucose, and lipid metabolism in peripheral tissues. In addition, their findings revealed that resveratrol treatment did not affect lipid content, activity, and the expression of AMPK, SIRT1, and PGC-1α in skeletal muscle [146]. Moreover, in a clinical study on healthy aged human subjects, Olesen et al. showed that resveratrol not only had no effect on improving mitochondrial oxidative function parameters and inflammation in skeletal muscle but, in some cases, it also reversed the beneficial effects of exercise. Interestingly, resveratrol did not have any impact on AMPK activation and SIRT1 and PGC-1α levels of skeletal muscle [147]. In addition, in a study on 17 subjects with well-controlled type 2 diabetes patients who were taking glucose-lowering drugs, resveratrol treatment for 30 days failed to improve insulin sensitivity in peripheral tissues and the liver. Although the function of mitochondria improved to some extent with the increase of respiratory capacity in skeletal muscle, resveratrol could not affect the increase of mitochondrial biogenesis and AMPK activity. They suggested that these results could probably be due to the interaction of resveratrol with metformin. Based on the findings of this study, it appears that resveratrol cannot act as a supplement to increase the efficacy of glucose-reducing drugs [148]. Furthermore, in a study on men with metabolic syndrome, Kjær et al. showed that taking resveratrol had no effect on the improvement of glucose metabolism and lipids. They showed that IMCL, the expression of genes involved in inflammation and mitochondrial function, and AMPK activity in skeletal muscle did not change [149]. In another study on 30 elderly adults with glucose intolerance, Pollack et al. showed that resveratrol had no effect on improving glucose tolerance and insulin sensitivity. Their deep transcriptomic study using RNA-Seq and ingenuity pathway analysis showed that genes involved in mitochondrial dysfunction and oxidative phosphorylation were disrupted during treatment with resveratrol in skeletal muscle. Additionally, although the content of mitochondria in skeletal muscle was increased, the morphology of mitochondria did not change [150]. Also, in a study on 13 men at high risk for type 2 diabetes, de Ligt et al. found that treatment with resveratrol for 30 days had no influence on improving insulin sensitivity and peripheral tissue glucose uptake. Although their results showed that resveratrol treatment could improve the ex vivo mitochondrial oxidative capacity function in skeletal muscle, however, mitochondrial abundance, mitochondrial in vivo function, and IMCL level in skeletal muscle were not affected by resveratrol. These results show that resveratrol can improve mitochondrial efficiency in skeletal muscle to some extent without affecting biogenesis; however, this effect of resveratrol on improving mitochondrial function is not enough to increase insulin sensitivity [151]. Similarly, in a clinical study on 38 obese and overweight subjects, Most et al. showed that the combined treatment of resveratrol and epigallocatechin gallate (EGCG) for 12 weeks, although increases the oxidative capacity of mitochondria and pathways of oxidative metabolism, but it had no effect on the accumulation lipid metabolites in skeletal muscle and did not improve insulin sensitivity in peripheral tissues [152].

According to the data of above-mentioned studies, conflicting results have been reported. While the results of some studies indicate a positive effect of resveratrol on skeletal muscle, some investigations report that resveratrol is ineffective. In general, the findings from human studies explain that resveratrol does not have any effect on improving insulin sensitivity in skeletal muscle. Also, contrary to in vitro and animal studies, most human studies reveal that resveratrol does not affect the AMPK and SIRT1 proteins in skeletal muscle. Furthermore, the results suggest that resveratrol does not affect the number and biogenesis of mitochondria in skeletal muscle; although in some studies, mitochondrial function was improved under the influence of resveratrol, and this improvement in mitochondrial function did not translate into enhanced insulin sensitivity in skeletal muscle. In addition, resveratrol did not affect the reduction of IMCL and inflammation of skeletal muscle. The exact details of the inconsistency between the results of human studies are not precisely known. One possibility for the difference in the results of the studies might be due to various doses and different intervention period of resveratrol used. The other potential reason for these conflicting results may be the difference in the baseline characteristics of the studied population, such as age, physical activity, and obesity level. In addition, the difference in study design may also be another possibility, because most of these studies have been conducted on various populations, such as healthy, obese, and aging individuals, postmenopausal women, diabetics, and people at high risk for diabetes. Another critical factor that may have an impact is an inter-individual variation in composition of people's gut microbiota because it has been shown to affect the metabolism of resveratrol and may cause discrepancies in the concentrations of different metabolites of resveratrol. In addition, there may be differences in the absorption of the active form of resveratrol from plasma between tissues. Consequently, to clarify the role of resveratrol on insulin sensitivity of skeletal muscles, further human studies are needed in the future.

## 6 Conclusions and future remarks

**Fig. 1:**
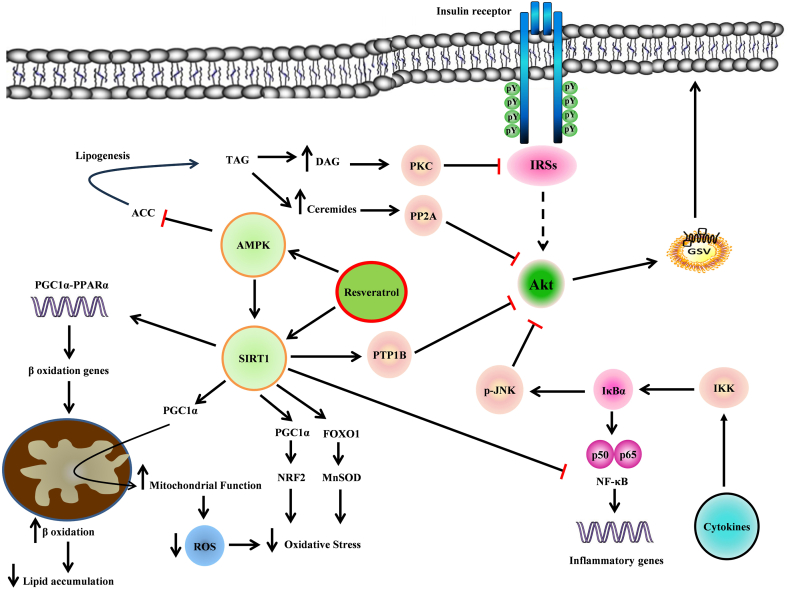
A schematic representation of the proposed mechanisms by which resveratrol improves insulin sensitivity in skeletal muscle.

This review has focused on the available information on the potential impact of resveratrol on insulin resistance in skeletal muscle. Based on the available data on cell culture and animal studies, resveratrol appears to have a beneficial role in improving insulin resistance in skeletal muscle by affecting different pathways such as insulin signaling, inflammation, oxidative stress, mitochondrial function, ER stress, and glucose and lipid metabolism (See Fig. 1). Furthermore, the current findings have suggested several mechanisms for the activity of resveratrol, which indicate that resveratrol may exhibit pleiotropic effects in the skeletal muscle to improve insulin resistance. However, one should be cautious about generalizing these results to humans because clinical trial studies' results are inconsistent. Therefore, more human studies are needed to clarify the effects of resveratrol on improving skeletal muscle insulin resistance.

SIRT1 and AMPK are activated by resveratrol. In complex with PGC1-α, SIRT1 activates fatty acid oxidation (via PPARα). In addition, AMPK activation reduces lipogenesis through inhibition of ACC leading to decrease reactive lipid intermediates such as DAG and ceramides and subsequently improvement of the insulin signaling. SIRT1 activation also reduces oxidative stress by modulating the levels of ROS, antioxidant enzymes, mitochondrial dysfunction in the cell through key transcription factors such as NRF2, FOXO, and PGC-1α. Inflammatory responses in skeletal muscle are also inhibited by SIRT1 activation through inhibition of NF-kB. SIRT1 activation also directly improve insulin signaling via inhibition of PTP1B. Finally, activation of SIRT1 leads to insulin-stimulated translocation of GLUT4 storage vesicles (GSVs) from intracellular storage pools to the plasma membrane resulting in improvement of insulin resistance. ACC, acetyl-CA carboxylase; DAG, diacyglycerol; FOXO1, Forkhead box protein O1; GSV, GLUT4 storage vesicles; IKK, inhibitor of β kinase; IRS-1, insulin receptor substrate-1; MnSOD, Manganese superoxide dismutase; JNK, Jun NH2-terminal kinase; NF-κB, Nuclear factor kappa-light-chain-enhancer of activated B cells; NRF2, Nuclear factor erythroid 2–related factor 2; PGC-1α, PPARα, Peroxisome proliferator-activated receptor α; Peroxisome proliferator-activated receptor γ coactivator 1-α; PP2A, protein phosphatase 2A; PKC, protein kinase C; PTP1B, Protein Tyrosine Phosphatase 1B; ROS, Reactive oxygen species; SIRT1, Sirtuin 1; TAG, triacylglycerol.

## Data availability statement

No data was used for the research described in the article.

## CRediT authorship contribution statement

**Arash Bahramzadeh:** Writing – review & editing, Writing – original draft, Data curation. **Kosar Bolandnazar:** Writing - review & editing. **Reza Meshkani:** Writing – review & editing, Visualization, Supervision, Investigation, Conceptualization.

## Declaration of competing interest

The authors declare that they have no known competing financial interests or personal relationships that could have appeared to influence the work reported in this paper.
